# Vertical transmission of *Salmonella enterica* serotype Paratyphi A leading to abortion

**DOI:** 10.1099/jmmcr.0.005127

**Published:** 2017-11-15

**Authors:** Shveta Sethi, Vikas Gautam, Kirti Gupta, Vanita Suri, Archana Angrup

**Affiliations:** ^1^​ Department of Medical Microbiology, Postgraduate Institute of Medical Education and Research, Chandigarh, India; ^2^​ Department of Histopathology, Postgraduate Institute of Medical Education and Research, Chandigarh, India; ^3^​ Department of Obstetrics and Gynaecology, Postgraduate Institute of Medical Education and Research, Chandigarh, India

**Keywords:** *Salmonella* Paratyphi A infection, abortion

## Abstract

**Introduction.** Enteric fever, caused by *Salmonella enterica* serotype Typhi (typhoid fever) or *S. enterica* serotype Paratyphi A, B or C (paratyphoid fever), is a major health problem in developing countries. Vertical transmission of *Salmonella* can cause miscarriage, still birth, preterm labour and neonatal sepsis. In the literature, many cases of vertical transmission of *S. enterica* Typhi from mother to foetus have been reported, but there are very limited studies showing vertical transmission of *S. enterica* Paratyphi.

**Case presentation.** Here, we report a rare case of *S. enterica* serotype Paratyphi A infection in a pregnant woman resulting in a spontaneous miscarriage. *S. enterica* serotype Paratyphi A was isolated from placental membrane in culture.

**Conclusion.** A high index of suspicion, along with timely cultures of relevant samples, like blood and stool, and timely initiation of antibiotic therapy in pregnancy could possibly save the lives of such foetuses.

## Abbreviation

PGIMER, Postgraduate Institute of Medical Education and Research.

## Introduction

Salmonellae are Gram-negative, nonsporulating, flagellate, facultative anaerobic bacilli. Genus *Salmonella* belongs to the family *Enterobacteriaceae* and consist of >2300 serotypes. Enteric fever, caused by *Salmonella enterica* serotype Typhi (typhoid fever) or *S. enterica* serotype Paratyphi A, B or C (paratyphoid fever) is a major public-health problem in developing countries [1].

The clinical presentation of human salmonellosis usually ranges from an asymptomatic, chronic carrier state to acute gastroenteritis, septicaemia and finally death. In the literature, many cases of vertical transmission of *S. enterica* Typhi from mother to foetus have been reported, but there are very limited studies showing vertical transmission of *S. enterica* Paratyphi A [2–4]. Enteric fever during pregnancy can result in miscarriage (65–80 %), stillbirth, preterm labour and neonatal sepsis [5]. Here, we report a rare case of transplacental transmission of *S. enterica* serotype Paratyphi A in a pregnant woman at 20+6 weeks of gestation resulting in a miscarriage.

## Case report

A 23-year-old married female (second gravida) with 20+6 weeks of pregnancy was admitted to the Department of Obstetrics and Gynecology, Postgraduate Institute of Medical Education and Research (PGIMER), Chandigarh, India, with complaints of fever, leaking per vaginum for 5 days, bleeding per vaginum for 2 days and abdominal pain for 1 day. She also gave a history of fever, intermittent abdominal pain and spotting (once in month) since the first trimester. She consulted a local private practitioner and was prescribed the antibiotic azithromycin (as a tablet) (500 mg,once daily for3 days). Then, she was referred to PGIMER, Chandigarh, by the local practitioner. On physical examination, her pulse rate was 110 beats min^−1^, her respiratory rate was 22 breaths min^−1^ and her temperature was 37.5 °C. Her blood pressure was 130/80 mmHg and remained within normal range during the other days of admission. Her cardiovascular system, respiratory system, gastrointestinal system and nervous system examinations were normal. She was a non-smoker and there was no history of any substance/alcohol abuse. She didn’t give any significant past and family history.

A per abdomen examination of the patient revealed a uterus of around 20 weeks of pregnancy with fetal parts palpable. The bimanual pelvic examination showed a patulous cervical os with altered blood coming out of it. Her routine blood investigations were sent for testing and abdomen ultrasound was performed. On the same day of admission, premature rupture of membranes with the spontaneous expulsion of an abortus of 450 g occurred. Placenta weighing 200 g was sent for culture to the Clinical Bacteriology Laboratory of the Department of Medical Microbiology, PGIMER, as well as for histopathological examination to the Department of Histopathology, PGIMER. Products of conception (abortus) were sent for autopsy. The patient remained admitted for 3 days and afterwards she was discharged with complete recovery.

## Investigations

The patient's abdomen ultrasound (at the time of admission) revealed severe oligoamnios. Routine blood investigations revealed haemoglobin of 11.8 g dl^−1^, a white blood cell count of 11 700 cells µl^−1^, 274×10^3^ platelets µl^−1^ and a coagulation profile (PT/aPTT/INR) that was within normal limits. Her serum electrolytes, total bilirubin and renal function tests (urea/creatinine) were also within normal limits. Her anti-human immunodeficiency virus (HIV) and anti-HCV antibodies and HBsAg were negative. VDRL test (Venereal disease Research laboratory) was nonreactive.

The Gram stain of placental membrane showed the presence of Gram-negative bacilli. On bacterial culture, *S. enterica* serotype Paratyphi A was isolated. The isolate was sensitive to nalidixic acid, ciprofloxacin, amoxicillin, ceftriaxone, co-trimoxazole and azithromycin. Histopathological examination of the placental membrane showed ‘acute chorioamnionitis’ suggestive of an infective aetiology. Fetal autopsy showed choroid plexus haemorrhage in the brain, a feature of prematurity.

## Diagnosis

There was no other likely cause of spontaneous miscarriage in this case. As *S. enterica* Paratyphi A was isolated from the placental membrane, it was considered to be the most likely cause.

## Treatment

Antibiotics injection cefixime (1 g, intravenously, twice daily) and injection metronidazole (100 ml, intravenously, three times daily) were started at the time of admission and continued for 3 days. After discharge, the patient was informed telephonically about the isolation of *S. enterica* Paratyphi A from the placental membrane and was called for follow up in the outpatient department.

## Outcome and follow-up

The patient didn’t come for review in the outpatient department; thus, we were not able to follow up her case further.

## Discussion

Although enteric fever is not common in developed countries, it still remains a persistent public-health problem in developing countries like India [6]. Human carriers are the only known reservoir of the disease. It is spread through ingestion of contaminated food and water. *S. enterica* Typhi is the most common aetiological agent and *S. enterica* Paratyphi A accounts for a very much lower number of cases (around 3–17 %) of enteric fever [7]. The actual burden of *S.* *enterica* Paratyphi in India and its characteristics related to enteric fever are poorly understood in the literature. According to one study, cases of enteric fever due to *S. enterica* Paratyphi A have shown an increasing occurrence in India [8]. In one survey from Delhi, there was a significant increase in the number of cases of *S. enterica* Paratyphi A from 6.5 % (1994) to 44.9 % (1998) [9]. A similar trend has been observed in Punjab state, with an increase from 14 to 40 % (2003–2007) [8]. The present case was also from Punjab.

In pregnant females, a similar incidence of infection with *Salmonella* (0.2 %) is seen when compared to the general population [10]. However, there is a lack of data about rates of chronic carriage of *S. enterica* Paratyphi A, and the extent to which it contributes to the infection. According to one study conducted by Khatri *et al.* in 2009 in Kathmandu, the paratyphoid fever carriage rate was 2 %, based on isolation from the gallbladder of cholecystectomy patients [11].

*Salmonella* has unfolded many defence mechanisms to elude the immune system of the host. Especially in pregnancy, there is a shift in immune status from type 1 (cell-mediated immunity) to type 2 (humoral immunity), which is more obvious at the maternal fetal interface [12]. Enhanced production of progesterone during pregnancy leads to suppression of cell-mediated immunity and increases the susceptibility to foodborne pathogens (like hepatitis E, salmonella, listeria, etc.) [13]. Different studies have shown that *S*. *enterica* Typhi and *S*. *enterica* Paratyphi A can be transmitted from the mother to foetuses/neonates by vertical transmission. Vertical transmission includes different modes like transplacental spread of the organism or as a result of bacteraemia of the mother during labour, or by the oral route during birth due to unintended faecal contamination of the birth canal at the time of delivery [14].

Salmonellae are well reported in abortion of sheep and bovine animals [15]. Fetal outcome appears to be related to the stage of pregnancy. It can range from spontaneous abortion to neonatal complications like sepsis, if contracted near the date of delivery. Untreated typhoid can cause a fetal loss rate of up to as high as 80 % [16]. In our case, isolation of *S. enterica* Paratyphi A from the placental membrane is consistent with the organism being the aetiological agent spread by crossing the maternal circulation into the placenta, in contrast to its isolation from vaginal swab, etc., where it may merely be a result of contamination. Histopathological Examination of placenta showing acute chorioamnionitis is further indicative of the infective aetiology. The mother was possibly a carrier or had concurrent typhoid infection at that time.

We carried out a PubMed search for cases of vertical transmission of *S. enterica* serotype Paratyphi A. Only two cases have been reported from India to the best of our knowledge. In one case report by Raveendran *et al.* in 2007 [4], both mother and neonate blood cultures were positive for *S.* *enterica* Paratyphi A and the neonate died of sepsis. In the second case report, by Mohanty *et al.* in 2009 [3], the preterm neonate was shown to be blood culture positive for *S.* *enterica* Paratyphi A and the baby survived, but the mother’s blood and stool cultures were negative for *Salmonella* (Table 1). In another case study, vertical transmission of *S.* *enterica* Paratyphi B was reported from South India [17].

**Table 1.** Cases of vertical transmission of *S. enterica* Paratyphi A from India

Case no.	Mother’s profile	Birth weight	Clinical features	Mode of transmission	Serotype	Outcome	Date of publication	Reference
1	Fever; blood culture – positive; delivery – Lower Segment Cesarean Section (LSCS)	1565 g	At birth – respiratory distress	Vertical	Paratyphi A	Death	March 29 2007	[4]
2	No history available; blood and stool cultures – negative; delivery – vaginal	1000 g	At birth – respiratory distress	Vertical	Paratyphi A	Discharged	July 6 2009	[3]

The limitations in our case were the inability to isolate *Salmonella* from the mother’s blood and stool samples due to loss of the patient to follow up.

To conclude, salmonellosis in the mother should be considered as a differential diagnosis, especially in those with a recent history of fever, to prevent fetal and maternal morbidity. Salmonellosis, in spite of being a dreadful illness in pregnancy, can easily be prevented by simple measures, such as hand hygiene, and avoidance of eating contaminated food, water and animal products. Only a high index of suspicion depending on the clinical presentation, along with proper laboratory diagnostics, can help in initiation of appropriate therapy in pregnancy to save the lives of these foetuses
